# The Association between Difficulty Seeing and Physical Activity among 17,777 Adults Residing in Spain

**DOI:** 10.3390/ijerph16214267

**Published:** 2019-11-03

**Authors:** Guillermo F. López-Sánchez, Igor Grabovac, Damiano Pizzol, Lin Yang, Lee Smith

**Affiliations:** 1 Faculty of Sport Sciences, University of Murcia, 30720 Murcia, Spain; 2 Department of Social and Preventive Medicine, Center for Public Health, Medical University of Vienna, 1090 Vienna, Austria; igor.grabovac@meduniwien.ac.at; 3 Italian Agency for Development Cooperation, Jerusalem 9135400, Israel; damianopizzol8@gmail.com; 4 Cancer Epidemiology and Prevention Research, Alberta Health Services, Calgary, AB T2S 3C3, Canada; lin.yang@ahs.ca; 5 Cambridge Centre for Sport and Exercise Science, Anglia Ruskin University, Cambridge CB1 1PT, UK; Lee.Smith@anglia.ac.uk

**Keywords:** vision problems, diabetic eye disease, physical activity, public health

## Abstract

This is the first representative population-based study exploring the association between difficulty seeing (i.e., low vision) and physical activity in Spain. Cross-sectional data from the Spanish National Health Survey 2017 were analysed (*n* = 17,777, ≥15 years; 52% females). Difficulty seeing was self-reported in response to the question ‘‘Do you have difficulty seeing?” The international physical activity questionnaire (IPAQ) short form was used to measure level of physical activity. Multivariable logistic regression was used to assess associations overall and by age group (15–49, 50–64, ≥65 years). Covariates included in the analysis were sex, age, education, marital status, use of glasses or contact lenses, cataracts, diabetes, obesity, hypertension, smoking and alcohol consumption. The overall prevalence of difficulty seeing was 11%, and the overall prevalence of participating in less than 600 metabolic equivalent (MET)-min/week of physical activity was 30.2%. After adjustment for covariates, difficulty seeing was associated with significantly higher odds of performing less than 600 MET-min/week of physical activity with the odds ratio (OR) = 1.222 (95% confidence interval = 1.099–1.357). Considering the impact on health and quality of life due to reduced physical activity in people with difficulty seeing, at least 600 MET-min/week of physical activity should be promoted to this population.

## 1. Introduction

In Europe, in 2010, there were 2,550,000 people who were blind and a further 23,800,000 with low vision resulting in 26,350,000 visually impaired individuals [1]. In addition, approximately 1.3 billion people globally live with distance or near vision impairment [1].

Influences on aetiology leading to vision impairment are multi-faceted, including increasing age [2], sex (women have been shown to be at higher risk than men) [3,4], higher lifetime ultra-violet exposure [5], systemic diseases (e.g., hypertension) [6,7], diabetes [7,8] or lower levels of education [3,9].

Vision impairment is a key barrier to the practice of physical activity in adults [10]. This makes it difficult for adults with vision problems to achieve the recommendation of at least 600 metabolic equivalent (MET)-min/week or 150 min of moderate-intensity activity over a week [11].

In fact, a small body of literature provides preliminary evidence that low vision is associated with lower levels of physical activity. A recent study, of older English adults (n = 6634), found that those with low vision were over twice as likely to be physically inactive compared with those with excellent vision [12]. Similarly, another study, with a sample of 6001 participants residing in the United States of America (USA), also suggests that visual impairment is associated with lower levels of physical activity [13]. Research has also found that children with low vision do less physical activity than those with good vision [14,15,16,17,18].

Considering the negative effects of visual impairment and physical inactivity on the quality of life and functional capacity of people, as well as the association between these two variables found in some previous studies, new population-based epidemiological studies exploring these links are needed. Specifically, in previously unreported countries, where the social and political context, physical environments (e.g. exposure to sunlight), and lifestyle behaviours, likely influencing these variables, are different

Several studies in the past two decades have investigated the prevalence of low vision in Spain. A cross-sectional observational study including 1155 elderly from the province of Cuenca estimated that the prevalence of visual impairment and blindness in the sample was 6.3% and 2.0%, respectively [19]. These findings were corroborated in a second study of 15,926 adults from Catalonia, as low vision was reported by 5.3% of women and 4.1% of men [20]. However, to the best of our knowledge, there are no representative population-based studies exploring the association between visual impairment and physical activity in Spain. Therefore, it is the aim of this study to explore the associations between the prevalence of visual impairment and the levels of physical activity in a Spanish population.

## 2. Methods

### 2.1. The Survey

Data from the Spanish National Health Survey 2017 were analysed. This survey was undertaken in Spain between October 2016 and October 2017. Details of the survey method are published elsewhere [21]. In brief, for the data collection, a stratified three-stage sampling was used in which the census sections were first considered, followed by the family dwellings and then an adult (15 years of age or more) was selected within each dwelling. The dwellings were selected by systematic sampling, and to select the person who had to complete the adult questionnaire, the random Kish method was used. The sample was representative of the adult population residing in Spain and consisted of 17,777 adults aged 15−69 years. The age group of adults ≥70 years was not considered in this study, as they did not complete the international physical activity questionnaire (IPAQ) short form. The IPAQ short form was developed for population surveillance of physical activity among adults aged 15−69 years. However, the use of the IPAQ with older and younger age groups is not recommended [22]. The method of data collection used was computer-assisted personal interviewing (CAPI), conducted in the homes of the selected participants. The interviewers, previously trained, completed the questionnaires with the information provided by the participants. All of them signed an informed consent form before responding to the survey questions. This research was conducted in accordance with the Declaration of Helsinki of 1961 (revised in Tokyo in 1989 and in Edinburgh in 2000).

### 2.2. Difficulty Seeing (Exposure)

Those who answered affirmatively to the question ‘‘Do you have difficulty seeing?” were considered to have difficulty seeing (i.e., visual impairment). In the case of those participants that used glasses or contact lenses, they were asked if they had difficulty seeing using their glasses or contact lenses.

### 2.3. Physical Activity (Outcome)

The IPAQ short form was used to measure physical activity. Total physical activity MET-min/week was calculated via the sum of walking + moderate + vigorous MET-min/week scores [22]. Participants were divided according to the guidelines for data processing and analysis of the IPAQ into (1) fewer than 600 MET-min/week and (2) at least 600 MET-min/week [20], equivalent to meeting the current physical activity recommendation. IPAQ has been validated in adult populations from different countries showing acceptable validity (ρ = 0.30, 95% CI: 0.23−0.36) and reliability (Spearman’s ρ = 0.81, 95% CI: 0.79−0.82) [23].

### 2.4. Covariates

Covariates were selected based on findings from the literature [12,13,24]. Sociodemographic covariates included in the models were sex, age, marital status and education. Education was categorised as ≤primary, secondary and ≥tertiary. Height and weight were self-reported. Body mass index (BMI) was calculated as weight in kilograms divided by height in meters squared. Obesity was defined as BMI ≥ 30 kg/m^2^. Participants who responded yes to the questions ‘‘Have you ever been diagnosed with diabetes/hypertension/cataracts?” were categorized as having diabetes/hypertension/cataracts. Those who answered affirmatively to the question ‘‘Do you use glasses or contact lenses?” were considered to use glasses or contact lenses. Smoking status was self-reported and categorised as never, current smoker or former smoker. Alcohol consumption in the last 12 months was self-reported and categorised as yes (any) or no (none).

### 2.5. Statistical Analysis

The statistical analysis was performed with SPSS 23.0 (IBM, Armonk, NY, USA). Differences in the prevalence of difficulty seeing by sample characteristics were assessed by Chi-squared tests. We conducted a multivariable logistic regression analysis to assess the association between difficulty seeing (exposure) and physical activity (outcome). Analyses were conducted for the overall sample and separately by age group (15–49, 50–64 and ≥65 years). All analyses were adjusted for sex, marital status, education, use of glasses or contact lenses, cataracts, diabetes, obesity, hypertension, smoking and alcohol consumption. The whole-sample analysis was also adjusted for age. All variables were included in the models as categorical variables. There were missing data only on the following variables: obesity (2.8%), smoking (0.1%) and alcohol consumption (0.1%). Complete case analysis was carried out. Results from the logistic regression analyses are presented as odds ratios (ORs) with 95% CIs. The level of statistical significance was set at *p* < 0.05.

## 3. Results

**Figure 1:**
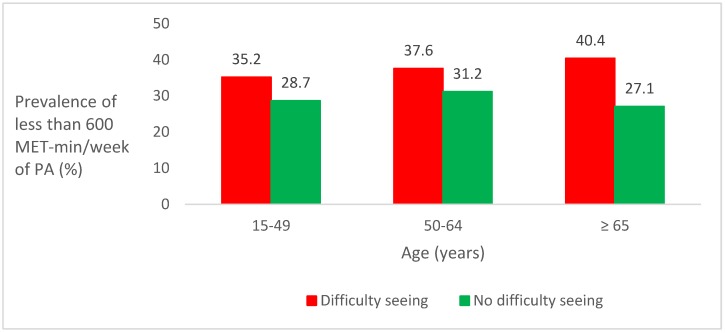
Prevalence of less than 600 metabolic equivalent (MET)-min/week of physical activity (PA) by age groups in those with and without difficulty seeing.

The sample consisted of 17,777 adults residing in Spain. The age range of the sample was 15−69 years, and the mean (standard deviation (SD)) age was 45.8 (14.1) years. 52% of the sample were female. The total average physical activity of the sample was 2263.7 ± 3222.9 MET-min/week (2025.2 ± 3374.7 MET-min/week in those with difficulty seeing and 2293.1 ± 3202.7 MET-min/week in those without difficulty seeing). The overall prevalence of difficulty seeing was 11%, and the overall prevalence of people doing less than 600 MET-min/week of physical activity was 30.2%. Overall, the prevalence of difficulty seeing among those doing less and more than 600 MET-min/week of physical activity was 13.4% and 9.9%, respectively. Overall, the prevalence of people doing less than 600 MET-min/week of physical activity among those with and without difficulty seeing was 36.9% and 29.4%, respectively. A linear increase in the prevalence of people doing less than 600 MET-min/week of physical activity was observed among those with difficulty seeing with increasing age. A decrease in this prevalence was observed among those without difficulty seeing who were older than 65 years (Figure 1).

Based on unadjusted estimates, ages between 50 and 64 years, female sex, being married, lower education, obesity, diabetes, hypertension, cataracts, smoking, no alcohol consumption and difficulty seeing were associated with significantly higher prevalence of doing less than 600 MET-min/week of physical activity (Table 1).

In the adjusted overall analysis, difficulty seeing was associated with significantly higher odds for those doing less than 600 MET-min/week of physical activity (OR = 1.222, 95% CI = 1.099–1.357) (Table 2). Lower age, female sex, being married, lower education, obesity, hypertension, smoking and no alcohol consumption were also associated with significantly higher odds of doing less than 600 MET-min/week of physical activity (Table 2). A final adjusted overall analysis was carried out considering only these previous variables associated with significantly higher odds of doing less than 600 MET-min/week of physical activity. In this case, difficulty seeing was also associated with significantly higher odds of doing less than 600 MET-min/week of physical activity (OR = 1.218, 95% CI = 1.097–1.353).

Age-stratified analyses showed that the association between difficulty seeing and physical activity was significant among those from 15 to 49 years old (OR = 1.200, 95% CI = 1.027–1.402) and among those older than 65 years (OR = 1.508, 95% CI = 1.124–2.023) (Table 3). This association was also significant when the analyses were adjusted only for the variables significantly associated with less than 600 MET-min/week of physical activity for 15–49 years old (OR = 1.198; 95% CI = 1.026–1.400) and older than 65 years (OR = 1.479, 95% CI = 1.105–1.979).

## 4. Discussion

To our knowledge, this is the first Spanish representative population-based study exploring the association between prevalence of difficulty seeing and physical activity. In this Spanish sample, the overall prevalence of participating in less than 600 MET-min/week of physical activity was 30.2%. These results are similar to those of the World Health Organization [25], which states that, globally, 27.5% of adults are insufficiently physically active. The overall prevalence of difficulty seeing in this Spanish sample was 11%, which is not high in comparison with a similar population-based study in the USA (33.8% uncorrected refractive error and 5.4% non-refractive visual impairment) [13]. The differences in these prevalence rates are likely to be due to differences in population characteristics, such as different ages [2], different sex distribution [3,4], different prevalence of systemic diseases (e.g., hypertension) [6,7], different prevalence of diabetes [7,8] or lower levels of education [3,9], as well as differences in the methods used between the studies.

The multivariable logistic regression showed that difficulty seeing was associated with 22.4% increased odds of doing less than 600 MET-min/week of physical activity. These results concur with other studies that have found negative associations between visual problems and physical activity in adults [12,13,26,27,28]. However, the association found in Spanish adults was weaker than in some previous studies. For example, a recent study, of older English adults (*n* = 6634), found that those with low vision were over twice as likely to be physically inactive compared with those with excellent vision [12]. Previous studies have indicated that the main barriers to an active lifestyle in people with difficulty seeing are lack of access to recreational and athletic programmes, lack of transport, lack of accessible exercise equipment, lack of help or encouragement in developing suitable and safe physical recreation skills and habits and activity limitations in walking [10,29]. Interventions that address these barriers to physical activity participation and that promote group physical activity [30] are required to increase activity levels in the Spanish population with difficulty seeing.

The main strengths of this study were the large representative sample and the use of a validated, reliable and internationally recognised questionnaire to measure physical activity. However, the results of this study should be considered within its limitations. The age group of adults ≥70 years was not considered, because the IPAQ short form is designed for the age range of 15–69 years. Assessment of difficulty seeing was self-reported, potentially introducing bias. This study did not include data about motor dysfunctions that could affect the amount of physical activity, and in consequence, it is recommended that this factor is considered in future studies. Moreover, the cross-sectional nature of the study means the direction of the association is not known. While we hypothesise that it is likely bidirectional, future longitudinal studies are needed to clarify the direction of causality.

## 5. Conclusions

The present study found a significant association between difficulty seeing and physical activity in a large sample of adults residing in Spain. Considering previous literature that has shown a negative impact on health and quality of life due to reduced physical activity in people with difficulty seeing, at least 600 MET-min/week of physical activity should be promoted.

## Figures and Tables

**Table 1:** Sample characteristics.

Characteristic	Category	*n*	Column%	*n* (%) Doing Less than 600 MET-min/week of PA ^a^		*p*-Value ^b^
Age (years)	15–49	10,089	56.8	2950	(29.2)	<0.001
	50–64	5977	33.6	1915	(32.0)	
	≥65	1711	9.6	501	(29.3)	
Sex	Female	9248	52.0	2990	(32.3)	<0.001
	Male	8529	48.0	2376	(27.9)	
Marital status	Married	10,010	56.3	3189	(31.9)	<0.001
	Not married	7767	43.7	2177	(28.0)	
Education	≤Primary	3324	18.7	1303	(39.2)	<0.001
	Secondary	9004	50.6	2712	(30.1)	
	≥Tertiary	5449	30.7	1351	(24.8)	
Obesity (BMI ≥ 30 kg/m^2^)	No	14,424	83.5	3992	(27.7)	<0.001
	Yes	2849	16.5	1137	(39.9)	
	Missing	504				
Diabetes	No	16,763	94.3	5007	(29.9)	<0.001
	Yes	1014	5.7	359	(35.4)	
Hypertension	No	14,633	82.3	4261	(29.1)	<0.001
	Yes	3144	17.7	1105	(35.1)	
Cataracts	No	17,113	96.3	5113	(29.9)	<0.001
	Yes	664	3.7	253	(38.1)	
Glasses or contact lenses	No	6653	37.4	1996	(30.0)	0.680
	Yes	11,124	62.6	3370	(30.3)	
Smoking	Never	8198	46.1	2355	(28.7)	<0.001
	Current	5071	28.5	1737	(34.3)	
	Former	4497	25.3	1265	(28.1)	
	Missing	11				
Alcohol Consumption	No	5371	30.2	1853	(34.5)	<0.001
	Yes	12,392	69.8	3505	(28.3)	
	Missing	14				
Difficulty seeing	No	15,828	89.0	4646	(29.4)	<0.001
	Yes	1949	11.0	720	(36.9)	

Notes: MET (Metabolic Equivalent of Task). BMI (body mass index). ^a^ Number (and percentage) of individuals with that sample characteristic who do less than 600 MET-min/week of physical activity. ^b^ *p*-value was calculated with Chi-squared tests.

**Table 2:** Association of difficulty seeing and other covariates with physical activity (outcome) estimated by multivariable logistic regression. (*n* = 17,777).

Characteristic		Less than 600 MET-Min/Week of Physical Activity	
Difficulty seeing	Yes vs. No	1.222 ***	(1.099, 1.357)
Age (years)	15–49	1.515 ***	(1.317, 1.742)
	50–64	1.378 ***	(1.211, 1.569)
	≥65	1.0	
Sex	Male vs. Female	1.272 ***	(1.187, 1.364)
Marital status	Married vs. Not married	1.236 ***	(1.154, 1.324)
Education	≤ Primary	1.763 ***	(1.587, 1.959)
	Secondary	1.235 ***	(1.140, 1.338)
	≥ Tertiary	1.0	
Obesity	Yes vs. No	1.605 ***	(1.469, 1.752)
Diabetes	Yes vs. No	1.013	(0.875, 1.173)
Hypertension	Yes vs. No	1.154 **	(1.050, 1.270)
Cataracts	Yes vs. No	1.253 *	(1.050, 1.495)
Glasses or contact lenses	Yes vs. No	1.023	(0.947, 1.106)
Smoking	Never	1.0	
	Current	1.394 ***	(1.287, 1.511)
	Former	1.013	(0.929, 1.105)
Alcohol consumption	Yes vs. No	1.190 ***	(1.104, 1.283)

Notes: Estimates are odds ratio (95% confidence interval). Models are adjusted for all variables in the table. * *p* < 0.05. ** *p* < 0.01. *** *p* < 0.001.

**Table 3:** Association of difficulty seeing and physical activity (outcome) by age groups estimated with multivariable logistic regression. (*n* = 17,777).

Age (Years)	Association between Difficulty Seeing and Less than 600 MET-Min/Week of Physical Activity (Outcome)	
15–49	1.200 *	(1.027, 1.402)
50–64	1.149	(0.972, 1.358)
≥65	1.508 **	(1.124, 2.023)

Notes: Estimates are odds ratio (95% CI). Models are adjusted for sex, marital status, education, obesity, diabetes, hypertension, cataracts, use of glasses or contact lenses, smoking and alcohol consumption. * *p* < 0.05, ** *p* < 0.01.
